# Hypertrophic olivary degeneration associated with bilateral vocal cord adductor dystonia

**DOI:** 10.1186/s12883-023-03123-8

**Published:** 2023-03-14

**Authors:** Mitchell Lycett, Cathy Kexin Cui, Dijana Dragicevich, Roger Harris, Karl Ng

**Affiliations:** 1 Department of Neurology, Royal North Shore Hospital, Reserve Road, St Leonards, New South Wales 2065 Australia; 2 Department of Speech Pathology, Royal North Shore Hospital, St Leonards, New South Wales Australia; 3 Department of Intensive Care Medicine, Royal North Shore Hospital, St Leonards, New South Wales Australia

**Keywords:** Oculopalatal tremor, Vocal cord, Dystonia, Myoclonus, Case report

## Abstract

**Background:**

Hypertrophic olivary degeneration (HOD) is a rare condition caused by lesions within the dentato-rubro-olivary pathway, resulting in ocular nystagmus and palatal myoclonus (oculopalatal tremor) but not usually dystonia. Dystonia is an uncommon association, and we present the first reported association of hypertrophic olivary degeneration with bilateral vocal cord dystonia.

**Case presentation:**

A 33 year old male presented initially with acute hydrocephalus on the background of previous ventriculoperitoneal (VP) shunting for previously treated medulloblastoma. After revision of the VP shunt, the patient developed progressive hiccups and stridor leading to respiratory failure requiring intubation. Ocular pendular nystagmus and palatal myoclonus at 3 Hz was observed. Flexible nasendoscopy (FNE) demonstrated bilateral tonic adduction of the vocal folds with 3 Hz coarse supraglottic, pharyngeal and palatal rhythmic myoclonus. MRI imaging demonstrated T2 hyperintensity within the bilateral inferior olivary nuclei consistent with stage 3 radiological HOD.

**Conclusions:**

Dystonia is a rarely reported phenomenon in HOD but is not unexpected with the inferior olivary nucleus implicated in dystonic disorders. We report the association of HOD with bilateral vocal cord adductor dystonia, a potentially life threatening condition.

**Supplementary Information:**

The online version contains supplementary material available at 10.1186/s12883-023-03123-8.

## Background

Hypertrophic olivary degeneration (HOD) is a rare condition caused by lesions disrupting the dentato-rubro-olivary pathway. It is associated with the development of ocular nystagmus and palatopharyngeal myoclonus described as oculopalatal tremor (OPT). We present a case of HOD related to distant medulloblastoma and its treatment, presenting additionally with bilateral vocal cord adductor dystonia, a previously unreported association.

## Case presentation

A 33 year old male presented acutely with headaches, dysarthria and increasing drowsiness in the context of previous ventriculoperitoneal (VP) shunting. The background was significant for medulloblastoma diagnosed at age 8 years, treated with surgical resection and adjuvant chemoradiotherapy. Other medical issues included hypopituitarism, radiation retinopathy and osteoporosis. A review 12 months prior had noted nocturnal stridor and palatal myoclonus which was presumed to be secondary to radiotherapy changes within the posterior fossa.

Initial imaging demonstrated hydrocephalus with a calcified and obstructed intra-abdominal portion of the VP shunt. A shunt revision was performed with radiological resolution of hydrocephalus. Days after the shunt revision, the patient developed hiccups and stridor progressing to respiratory failure requiring intubation then tracheostomy formation. Flexible nasendoscopy (FNE) demonstrated bilateral adducted midline vocal folds with no change in positioning in response to inspiration, expiration or vocalisation suggestive of dystonia. FNE also observed 3 Hz coarse supraglottic, pharyngeal and palatal rhythmic myoclonus (Videos 1 and 2). The patient later developed ocular pendular nystagmus and rhythmic facial muscle twitching consistent with the syndrome of oculopalatal tremor (OPT). Repeat electroencephalograms (EEGs) did not demonstrate epileptiform activity, nor a cortical electrical correlate for the OPT. There was no response in the OPT or tonic positioning of the vocal folds to trials of benzodiazepines, sodium valproate, levetiracetam or pulse steroids.

**Fig. 1:**
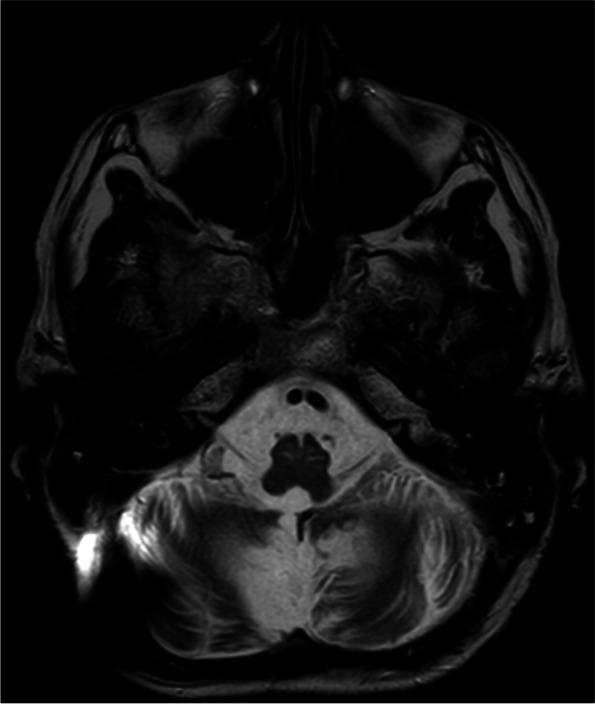
T2-weighted MRI demonstrating hyperintensities within the bilateral inferior olivary nuclei. The absence of inferior olivary nuclei hypertrophy is consistent with stage 3 radiological HOD [1]. Additional findings include cerebellar gliosis and atrophy secondary to previous posterior fossa surgery and radiotherapy

MRI imaging demonstrated T2 hyperintensity within the bilateral inferior olivary nuclei consistent with progressed HOD (Fig. 1). It also showed known cerebellar atrophy and gliosis secondary to previous surgery and radiotherapy, and bilateral calcified subdural haematomas complicating long-term VP shunting. There were no abnormalities identified in the basal ganglia. Previous MRI imaging after the completion of childhood medulloblastoma treatment did not show any evidence of developing HOD.

FNE was repeated at 6 weeks with unchanged dystonic midline positioning of the vocal folds. In discussion with the patient and family, the tracheostomy was decannulated after a period of 10 weeks and the patient was palliated. Laryngeal EMG was not performed as it would not change the palliative management.

## Discussion and conclusions

Hypertrophic olivary degeneration (HOD) is a well described clinical syndrome resulting from deafferentation of the inferior olivary nucleus, usually secondary to lesions within the dentato-rubro-olivary pathway, otherwise known as the Guillain-Mollaret triangle [1, 2]. This classically presents with synchronous ocular pendular nystagmus and palatal tremor at a frequency of 2–3 Hz [1, 3]. Involvement of other muscles derived from the branchial arches such as the facial muscles, larynx and diaphragm can occur, as was demonstrated in the described case.

The syndrome of HOD is associated with a variety of secondary causes including ischaemic or haemorrhagic strokes, demyelination and posterior fossa tumours [1, 3]. When secondary to posterior fossa tumours, the syndrome usually develops at the time of tumour diagnosis or within 1 year of surgical resection [1, 4]. The late presentation in the described case, occurring 25 years after initial surgery and adjuvant chemoradiotherapy, is atypical and symptom onset may be related to the late effects of radiotherapy. An association between HOD and raised intracranial pressure such as due to hydrocephalus has not been described, although the acute illness is suspected to have augmented the HOD presentation in this case. The VP shunt revision could have generated a state of relative intracranial hypotension, with vocal cord palsy previously reported with iatrogenic intracranial hypotension [5]. HOD secondary to intracranial hypotension has not been described.

Vocal cord paralysis has been reported in the context of both intracranial hypertension and hypotension. This is typically in the paediatric population with Chiari malformations and is thought to be a result of vagal or recurrent laryngeal nerve stretch injuries [6]. A tight midline position of the bilaterally adducted vocal folds with obliteration of the glottic space is unusual for bilateral vocal cord paralysis, with the vocal folds typically assuming a paramedian rather than the midline position seen in recurrent laryngeal nerve palsies and mid-abducted “cadaveric” position seen in high vagal and brainstem lesions [7, 8]. In this case, the midline positioning of the vocal folds, as well as the presence of laryngeal myoclonus and the synchronous onset with other features of HOD, suggests that the pathologically adducted vocal fold positioning was dystonic in origin rather than secondary to vocal cord palsy. The presence of supraglottic and laryngeal myoclonus suggests that vagal innervation is at least partially intact. Therefore, if the vocal cord positioning was thought to be secondary to a vagal or laryngeal nerve palsy, then some movement of the laryngeal folds with phonation would be expected. This is not demonstrated in Video 1 and provides further evidence for a dystonic pathology. The absence of dilatation of the piriform sinuses also provides evidence against vocal cord paralysis as the cause of the bilaterally adducted vocal cord position.

Dystonia is a rarely reported association with HOD, with previous cases describing limb dystonia only [9, 10]. We report the first case of HOD associated with bilateral dystonic adduction of the vocal folds. This association is not entirely unexpected, with mouse models implicating the inferior olivary nucleus in dystonic disorders [11]. Drug-related dystonia related to dopaminergic receptor blocking agents such as antipsychotics can also manifest as acute laryngeal dystonia, however the patient in this case did not receive any such causative agents [12]. Additional aetiologies of laryngeal dystonia such as genetic causes could theoretically also be unmasked by the patient’s acute illness but the absence of a relevant family history and concurrent MRI changes consistent with HOD, make HOD a more compelling cause.

The treatment of HOD is usually symptomatic, with a variable response to medical therapies such as benzodiazepines or anticonvulsants [1]. Unilateral HOD typically slowly improves but bilateral HOD is associated with a poor prognosis [1]. Laryngeal dystonia is often poorly responsive to benzodiazepine and anticonvulsant therapy, with no significant response seen in the presented case [13]. Botulinum toxin may be effective in adductor forms of laryngeal dystonia, and could have been performed if tracheal decannulation was the only goal in this case [13].

## Supplementary Information

**Additional file 1:** **Video 1.** Flexible nasendoscopy demonstrating bilaterally adducted midline vocal folds with laryngeal and pharyngeal myoclonus at 3 Hz.

**Additional file 2:** **Video 2.** Palatal myoclonus at 3Hz.

## Data Availability

Not applicable.

## Abbreviations

HOD: Hypertrophic olivary degeneration
OPT: Oculopalatal tremor
VP: Ventriculoperitoneal
FNE: Flexible nasendoscopy
EEG: Electroencephalogram

## References

[1] Wang H, Wang Y, Wang R (2019). Hypertrophic olivary degeneration: a comprehensive review focusing on etiology. Brain Res.

[2] Murdoch S, Shah P, Jampana R (2016). The Guillain-Mollaret triangle in action. Pract Neurol.

[3] Borruat FX (2013). Oculopalatal tremor. Curr Opin Neurol.

[4] Hirano M, Hatzoglou V, Karimi S, Young RJ (2015). Hypertrophic olivary degeneration resulting from posterior fossa masses and their treatments. Clin Imaging.

[5] Aytuluk HG, Aktas O (2018). Vocal fold paralysis due to intracranial hypotension following spinal anesthesia. Anaesthesist.

[6] Arora N, Juneja R, Meher R, Bhargava E (2016). Bilateral vocal cord palsy with Arnold Chiari malformation: A rare case series. J Clin Diagn Res.

[7] Dankbaar JW, Pameijer FA (2014). Vocal cord paralysis: anatomy, imaging and pathology. Insights Imaging.

[8] Wareing MJ, Millard R, Siddiqui J. Chapter 32. Vocal Cord Paralysis. In: Lalwani AK. eds. CURRENT Diagnosis & Treatment in Otolaryngology—Head & Neck Surgery, 3e. McGraw Hill; 2012. p. 573–579.

[9] Yang J, Yang J, Li B, Bao L (2020). Transient palatal tremor and action induced foot and leg dystonia due to hypertrophic olivary degeneration: a case report. J Stroke Cerebrovasc Dis.

[10] Kim HJ, Cho YJ, Cho JY, Hong KS, Jeon BS (2008). Choreodystonia in a patient with hypertrophic olivary degeneration after pontine tegmental hemorrhage. Mov Disord.

[11] White JJ, Sillitoe RV (2017). Genetic silencing of olivocerebellar synapses causes dystonia-like behaviour in mice. Nat Commun.

[12] Collins N, Sager J (2018). Acute laryngeal dystonia: a drug-induced respiratory failure related to antipsychotic medications. J Neurol Neuromedicine.

[13] Simonyan K, Barkmeier-Kraemer J, Biltzer A (2021). Laryngeal dystonia. Neurology.

